# Characterization of Gelatin-Sodium Alginate Complex Coacervation System

**DOI:** 10.4103/0250-474X.56033

**Published:** 2009

**Authors:** Ujwala A. Shinde, Mangal S. Nagarsenker

**Affiliations:** Bombay College of Pharmacy, Kalina, Santacruz (East), Mumbai-400 098, India

**Keywords:** Complex coacervation, phase separation, gelatin, sodium alginate

## Abstract

A gelatin and sodium alginate complex coacervation system was studied and an effect of pH and colloid mixing ratios on coacervation was investigated. A colloid mixing ratio at which optimum coacervation occurred varied with the coacervation pH. Viscometric, turbidity and coacervate dry yield investigations were used to investigate optimum conditions for complex coacervation. Optimum coacervation occurred at pH 3.5 at a gelatin sodium alginate ratio 4:1. Coacervate and equilibrium fluid was analyzed for gelatin and sodium alginate contents and yields calculated on the basis of chemical analysis showed that optimum coacervation occurred at 25% sodium alginate fraction at pH 3.5.

Complex coacervation-phase separation is one of the first techniques used in process of microencapsulation. Coacervation was first described by Bungenberg de Jong as a process of flocculation or separation of liquids from a solution in which one phase rich in colloid is called as coacervate phase and the other containing very little colloid called equilibrium fluid[1].

Complex coacervation the most widely studied system contains two or more colloidal solutes. The process depends on the formation of salt bonds associated with macromolecules to bring about coacervation. Gelatin-acacia system has been characterized by Bungenberg de Jong through measurements of viscosity, turbidity, coacervate volume and electrophoresis[1]. Other combinations, which have been studied are carbapol-gelatin[2], pectin-gelatin[3], gelatin-gelatin[4], sodium alginate-gelatin[5], sodium carboxymethyl cellulose-gelatin[6,7], albumin-alginic acid[8] and albumin-acacia[9].

Complex coacervation can be induced in systems containing cationic and anionic hydrophilic colloids; sodium alginate a water soluble seaweed gum is widely used in various dosage forms and in the area of drug delivery systems. Gelatin, an amphoteric protein, which is positively charged below its isoeletric point and is expected to form complex coacervate with sodium alginate, which will give negative charges at lower pH. Arneodo *et al,* investigated the complex coacervation of gelatin with gum arabic, sodium alginate and sodium phosphate in which coacervation was induced by dilute acetic acid. The systems were characterized through the measurements of viscosity and solid contents of coaceravate[5]. Present study will provide an insight into factors affecting properties of gelatin sodium alginate complex coacervate system and hence microcapsules.

Gelatin bacteriological grade, alkali processed Type B material (180 bloom) was obtained from Loba Cheme, Mumbai, India. Sodium alginate, medium viscosity pharmaceutical grade was purchased from Loba Cheme, Mumbai, India. All other regents were of analytical reagent grade.

For complex coaceravtion, binary mixtures of gelatin and sodium alginate were prepared by dispersing gelatin and sodium alginate separately in distilled water, allowed to hydrate for 1 h followed by mixing. Coacervate were prepared in 15 ml calibrated centrifuge tubes at controlled temperature by adding dilute hydrochloric acid to the colloid solution, stirring with a cyclomixer. The coacervate mixtures were allowed to equilibrate before being subjected to further evaluation. The ratios of gelatin:sodium alginate studied were 10:1, 6:1, 4:1, 2:1 and 1: 1.

A series of gelatin and sodium alginate mixtures (total polymer concentration 0.2% w/v) were prepared by dispersing them in distilled water and pH was adjusted in the range 2.5 to 7.0. The absolute viscosity of the equilibrium fluid of coacervate mixtures was determined in triplicate using U tube viscometer at controlled temperature.

Turbidity of gelatin sodium alginate mixtures (0.05% w/v total colloid concentration) was measured at pH range 1.5 to 4.5 in duplicate at 600 nm (Shimadzu UV-160A UV/Vis spectrophotometer) in 1 cm cuvetts against distilled water.

Coacervates were prepared using standard procedure in calibrated 15 ml centrifuge tubes at 3% w/v total colloid concentration and adjusting pH in the range 2.5 to 4.0. The coacervate volume was measured, the equilibrium fluid was decanted and the coacervate wet weight was measured. Wet coacervate was dried at 60° till constant dry weight was obtained. The solution of coacervate gel was prepared by dissolving the coacervate gel in 0.2N sodium hydroxide. This solution and equilibrium fluid were diluted appropriately with distilled water and analyzed for gelatin by Biuret assay and sodium alginate by phenol-sulphuric acid assay.

The percent specific viscosities were calculated from the measured absolute viscosity values. The changes in percent specific viscosities of a series of gelatin-sodium alginate mixture as a function of the colloid mixing ratio are shown fig. 1. In the mixture of gelatin and sodium alginate at pH 1.5, additive behavior of viscosity was not observed because at such low pH, sodium alginate precipitates as alginic acid which is water insoluble resulting in decrease in viscosity. In addition, as its pka value is in the range of 3.4 to 4.4. At such low pH, it mainly exists in unionized form indicating no gelatin-sodium alginate interaction[10].

**Fig 1:**
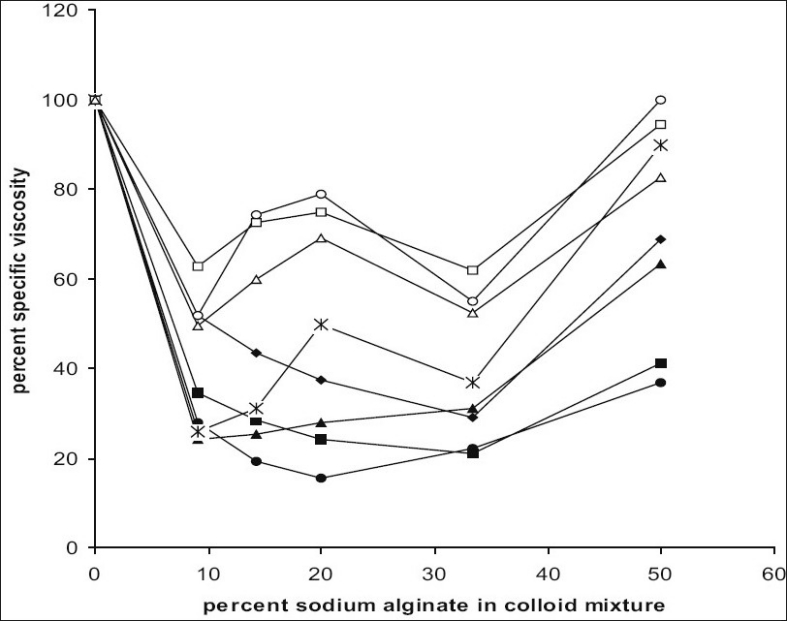
Effect of % sodium alginate content in colloid mixture on % specific viscosity. pH 2.5 (–◆–), pH 3.0 (–■–), pH 3.5 (–●–), pH 4.0 (–▲–), pH 4.5 (–Ж–), pH 5.0 (–○–), pH 6.0 (–□–), pH 7.0 (–Δ–)

Coacervation was clearly evident at all colloid mixing ratios over pH range 2.5 to 4 and minimum values of viscosity were noted. In this pH range, sodium alginate was negatively charged and gelatin was positively charged, resulting in electrostatic interaction and hence complex coacervation. As the coacervation pH was raised from 2.5 to 4, the number of anionic charges on sodium alginate was increased while net positive charge on gelatin was decreased until it reached to zero at its isoelectric point (pH 4.7). As a result of this; decreasing amount of sodium alginate was required for an equivalent interaction to occur with gelatin, resulting in coacervation. Thus, a shift in position of minima of curves was observed with pH change. Fig. 1 shows that at pH 2.5, sodium alginate 33.33 % of total polymer gave optimal coacervation indicated by minimum in the curve, whereas at pH 4 the optimal coacervation occurred at 9.09% sodium alginate concentration.

Percent specific viscosity of coacervate system was increased with rise in pH from 4.5 to 7. In this pH range solution remained clear, indicating no coacervation at all colloid mixing ratios. This rise in viscosity indicated that there was sufficient interaction between the two colloid to cause rise in viscosity but not for coacervation to occur. This could be attributed to two factors: the degree of dissociation of carboxylic groups on sodium alginate and the number of positive charges on the gelatin molecule. At the isoelectric point, there exist an equal number of anionic and cationic charges on gelatin molecule, thus an ionic interaction with anionic sodium alginate molecule may result in the formation of negatively charged soluble gelatin sodium alginate complex. Also the viscosity of sodium alginate is slightly higher near neutrality because of repulsive effects of negatively charged carboxyl groups that extend the polymer chain and increase its water binding capacity[10]. However, maximum coacervation (i.e. minimum viscosity) occurred at pH 3.5 and 20% sodium alginate concentration. At this pH, sodium alginate and gelatin were oppositely charged and thus, this was the value of pH which was most favorable for complex coacervation of gelatin and sodium alginate.

Turbidimetry measurements can be used to study complex coacervation at low concentration of 0.05% w/w. The pH range for coacervation of gelatin and sodium alginate when estimated by turbidimetry measurements was 2.5 to 4.5 was in agreement with viscometric data (fig. 2). From the results of viscosity and turbidity measurements, it was seen that there was a corresponding equivalent mixing proportion at each pH. Coacervation was only vigorous at pH values, which lie too close to the extremities of this pH range. At pH 4.5 coacervation was only slight indicated by hazy appearance of system and at pH 2.0 it was even absent.

**Fig. 2:**
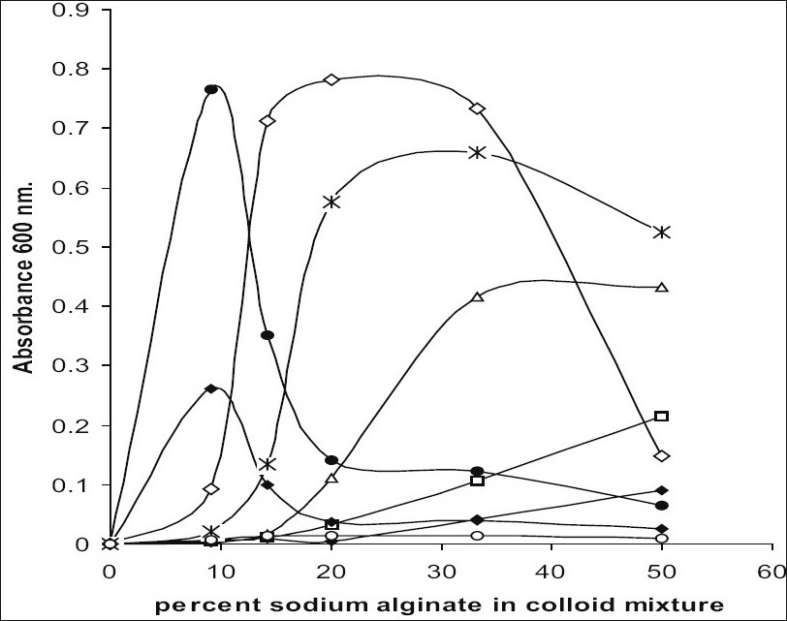
Effect of % sodium alginate content in colloid mixture on turbidity. pH 1.5 (–◆–), pH 2.0 (–□–), pH 2.5 (–Δ–), pH 3.0 (–Ж–), pH 3.5 (–◊–), pH 4.0 (–●–), pH 4.5 (–+–), pH 5.0 (–○–)

The coacervate composition and electrical charge on the droplets are known to be dependent on colloid type, colloid mixing ratio, total colloid concentration, coacervation pH and presence of electrolyte[1]. Dry coacervate weight has been adopted as a measure of coacervate yield to determine the maximum degree of coacervation[3].

The effect of colloid ratio on coacervate dry yield is shown in fig. 3. It was clearly evident that as the percentage of sodium alginate in coacervate mixture was increased, yield also increased till it reached to optimum value after which it decreased. At higher fraction of sodium alginate, colloid solution became very viscous which lead to poor separation of coacervate and hence decreased yield. It was noted that increase in the pH resulted in a shift of optimal mixing ratio towards a lower sodium alginate fraction i.e. peak yield occurred at 50% sodium alginate at pH 2.5 but at pH 4 peak yield was obtained at 20% sodium alginate concentration. Thus, for each colloid ratio the maximum coacervate yield was obtained at one pH value which was optimum for particular ratio. The maximum yield occurred at 20% sodium alginate concentration at pH 3.5.

**Fig. 3:**
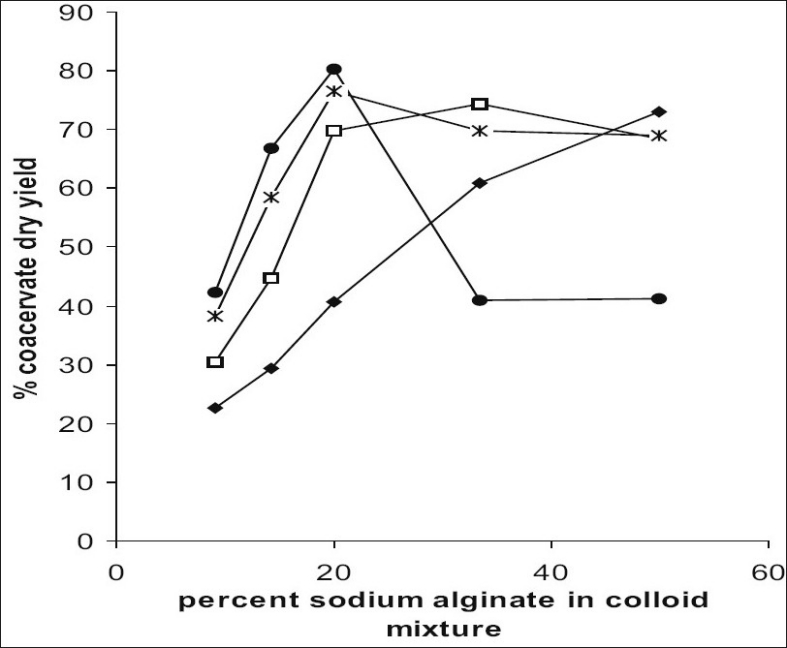
Effect of % sodium alginate content in colloid mixture on percent coacervate dry yield. pH 2.5 (–◆–), pH 3.0 (–□–), pH 3.5 (–Ж–), pH 4.0 (–●–).

Complex coacervation phase separation results in distribution of the dissolved colloid into coacervate phase and equilibrium fluid phase[1]. Physical methods of characterization include viscosity, turbidity and coacervate dry yield did not give the exact concentration of individual colloid in caocervate phase and equilibrium fluid phase[6,7]. Therefore, chemical method of analysis of gelatin and sodium alginate in both phases was investigated.

A gelatin and sodium alginate content estimated by biuret method and phenol sulphuric acid method was used for construction of ternary phase diagram. Phase diagrams of the gelatin sodium alginate complex coacervation system at pH values 2.5, 3.0, 3.5, and 4 are shown in fig. 4. Equilibrium fluid data is shown only for pH 3.5 and data for other pH values are not shown because of closeness of values.

**Fig. 4:**
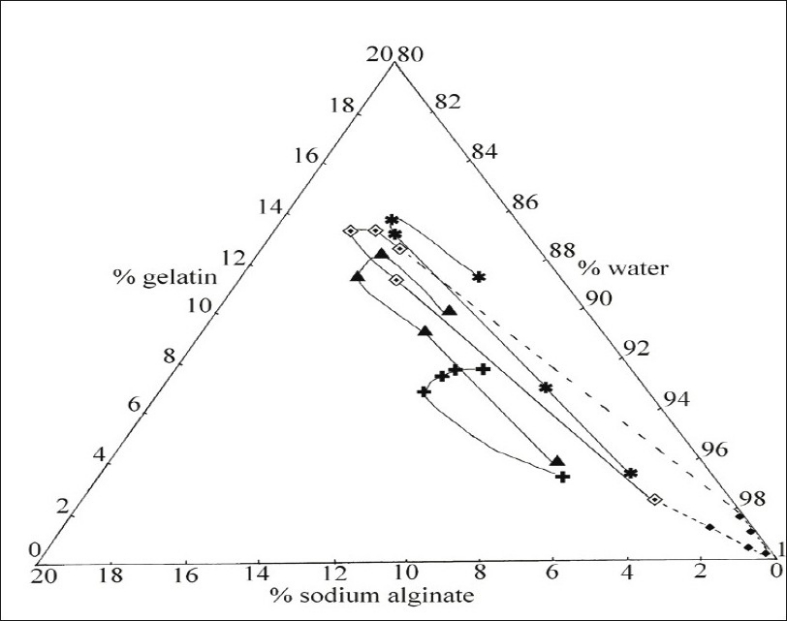
Effect of pH and colloid concentration on ternary phase diagram complex coacervation system. Colloid concentration at pH 4 (–✱–), pH 3.5 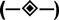, pH 3 (–▲–), pH 2.5 (–✚–) in coacervate and pH 3.5 (–◆–) in equilibrium fluid

The ternary phase diagram obtained for the optimum pH of 3.5 exhibited a drop-shaped two-phase region located in the water corner. This shape is characteristic of complex coacervation, which generally takes place between oppositely charged molecules. Complex coacervation involves electrostatic interaction between anionic sodium alginate and cationic gelatin. The increase in coacervation pH from 2.5 to 4 increased the number of anionic charges on sodium alginate while net positive charge on gelatin was decreased. Hence, less sodium alginate was required for an equivalent interaction with gelatin as pH was raised. This resulted in decrease in sodium alginate content of coacervate, which was indicated by displacement of the region of phase separation towards lower sodium alginate percentage in phase diagram.

Changes in colloid composition of complex coacervates and equilibrium fluid of isohydric mixtures at pH 3.5 are shown in fig. 5. Composition of total colloid mixture is represented by the broken line. Intersection of the coacervate curve and the equilibrium fluid curve occurred at the equivalent mixing ratio.

**Fig. 5:**
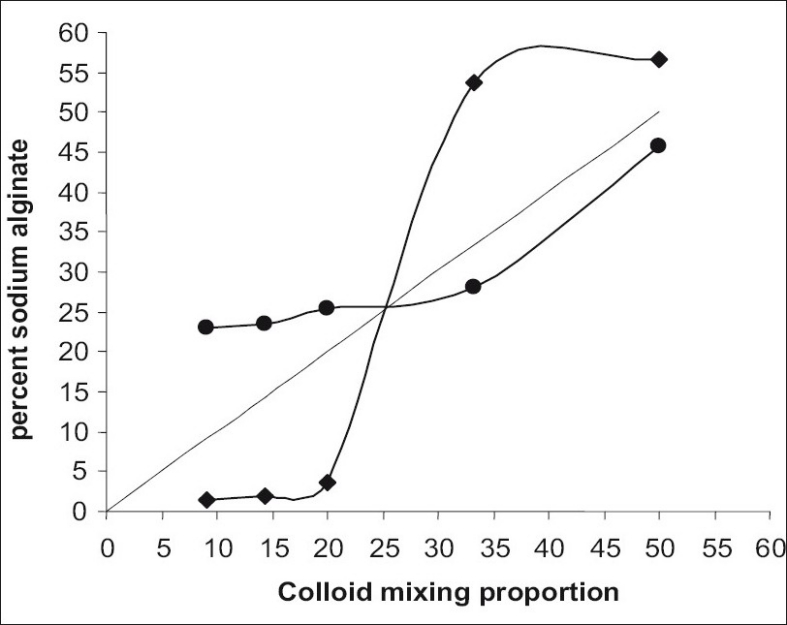
Effect of colloid mixing ratio on % sodium alginate concentration in colloid mixture. Percent sodium alginate in coacervate (–●–), percent sodium alginate in equilibrium fluid (–◆–)

At equivalent mixing ratio composition of coacervate, equilibrium fluid and total mixture were the same. The equivalent mixing ratio for gelatin-sodium alginate complex coacervate system was found to be 25% w/v of sodium alginate fraction in colloid mixing ratio. The electrophoretic reversal of charge point lies at or nearly at, the equivalent mixing proportion[1]. At electrical equivalence pH and mixing ratio where the charge on the two colloids are equal and opposite, attraction forces between the charged colloids saturate each other leading to intense interaction and highest degree of coacervation[4].

Maximum coacervation occurred at a colloid-mixing ratio very close to equivalent mixing ratio. At the other colloid mixing ratios where the charges were no longer balanced, there was a reduction in the interaction between the oppositely charged colloids and thus a lesser degree of coacervation.

The results obtained in present study support the hypothesis of complex coacervation phenomenon between gelatin and sodium alginate. The effects of pH and colloid ratio were demonstrated by viscometric, turbidimetric and coacervate yield. The colloid ratio gelatin:sodium alginate (4:1) at pH 3.5 showed maximum degree of coacervation, which provides a basis for design of microcapsules.
